# Native flexibility of structurally homologous proteins: insights from anisotropic network model

**DOI:** 10.1186/s13628-017-0034-9

**Published:** 2017-01-31

**Authors:** Ranja Sarkar

**Affiliations:** School of Computational and Integrative Sciences, Jawaharlal Nehru University, New Delhi, 110 067 India

**Keywords:** Anisotropic network model, Fibronectin, Flexibility, Spring constant

## Abstract

**Background:**

Single-molecule microscopic experiments can measure the mechanical response of proteins to pulling forces applied externally along different directions (inducing different residue pairs in the proteins by uniaxial tension). This response to external forces away from equilibrium should in principle, correlate with the flexibility or stiffness of proteins in their folded states. Here, a simple topology-based atomistic anisotropic network model (ANM) is shown which captures the protein flexibility as a fundamental property that determines the collective dynamics and hence, the protein conformations in native state.

**Methods:**

An all-atom ANM is used to define two measures of protein flexibility in the native state. One measure quantifies overall stiffness of the protein and the other one quantifies protein stiffness along a particular direction which is effectively the mechanical resistance of the protein towards external pulling force exerted along that direction. These measures are sensitive to the protein sequence and yields reliable values through computations of normal modes of the protein.

**Results:**

ANM at an atomistic level (heavy atoms) explains the experimental (atomic force microscopy) observations viz., different mechanical stability of structurally similar but sequentially distinct proteins which, otherwise were implied to possess similar mechanical properties from analytical/theoretical coarse-grained (backbone only) models. The results are exclusively demonstrated for human fibronectin (FN) protein domains.

**Conclusions:**

The topology of interatomic contacts in the folded states of proteins essentially determines the native flexibility. The mechanical differences of topologically similar proteins are captured from a high-resolution (atomic level) ANM at a low computational cost. The relative trend in flexibility of such proteins is reflected in their stability differences that they exhibit while unfolding in atomic force microscopic (AFM) experiments.

## Background

Proteins are often subjected to mechanical pressures in the cell, as a consequence of their interactions with other cellular biomolecules for example, within muscle fibres, in microtubules and molecular motors. These mechanical forces are functionally relevant and led to evolution of the mechanical behavior of proteins in order to fit their biological functions [1]. Revelations from single-molecule AFM and optical-tweezer pulling experiments aided researchers to investigate the response of proteins to external forces directed along lines connecting particular residue pairs in the proteins [2–4]. Unfolding forces in different directions have been experimentally measured for ubiquitin and green fluorescent protein [5, 6] and considerable differences have been observed between mechanical resistances of the same molecule to different deformation directions, which reflect the anisotropic property of the molecule. However, these experiments can be complicated with time limitations and to examine the mechanical stability of proteins can be quite difficult.

Some theoretical studies have been done to gain molecular insights on the origins of anisotropic responses of proteins and the role of secondary structure composition in determining the unfolding pathways, in particular molecular dynamics (MD) simulations [7–10]. Steered MD and equilibrium MD have been performed on several small globular proteins to understand the structural features responsible for a particular unfolding pathway as opposed to the other and thereby, for the mechanical stability of proteins. MD simulations did highlight the relevance of native contact topology in the unfolding pathways. The impact of native contacts on the mechanical behavior of proteins could as well be investigated using simple network models such as ANM which are computationally inexpensive [11].

Here I present an approach that is computationally fast and analytically simple. An earlier result published by E Eyal and I Bahar effectively demonstrated the use of an analytical method (coarse-grained ANM) to construct a complete map of the mechanical response of a protein to all possible deformation directions in the protein [12]. However, their methodology could not explain the different responses exhibited by structurally homologous but sequentially different structures. An interesting example is the heparin-binding and integrin-binding segments of the human FN protein [13]. The lack of residue specificity in their model does not allow its applicability to proteins wherein the sequence identity (and not the native fold) dominates the mechanical behavior.

In the present study, the previous model has been extended by incorporating all heavy atoms instead of the C_α_ atoms only (coarse-graining [12]). Results from an all-atom ANM are illustrated which explain the distinct responses of N-C termini residue pairs under tension of structurally identical FN domains and hence, the relative flexibility of the two domains. An all-atom representation of ANM strictly means that using this model, the normal modes are calculated for all the heavy atoms in the native crystal structure of the protein, as available from the protein data bank. The atomic coordinates are used from the fibronectin crystal structure of PDB code 1fnh (resolution: 2.8 Å) [13]. The model at its atomistic level (and not the residue level) is used to define two kinds of spring constants as observables (or theoretical measures), one is associated with uniaxial deformation along a residue pair (as defined in [12]) and the other is associated with the overall mobility of atoms (heavy) in the protein as indicated by the normal modes. The normal modes dictate the protein dynamics near its equilibrium (native) state and their spring constants correlate well with the experimental measures. This approach highlights the ease of extracting reliable values for the proposed spring constants through computations as inexpensive as ANM.

The methods for calculation of the two spring-constant types from the normal modes of the protein are described in the next section, followed by the results obtained in the succeeding section. The reaction coordinate (N-C termini direction) defined by single-molecule AFM experiments [14] for the FN domains has been assessed in the present work and the results are summarized in the concluding section.

## Methods

ANM provides a simple two-parameter harmonic interaction potential to generate normal modes of vibration for a protein structure [15]. The protein is modeled as an elastic network of nodes and springs, wherein the nodes of the network are defined by either Cα-atom (crude residue-level description) or heavy atoms (finer all-atom resolution) in the input structure (X-ray or NMR). The springs are representative of effective (bonded as well as non-bonded) interactions between the pairs of residues/atoms. Typically, only nodes (connected by springs) separated by a distance smaller than a cutoff ${R}_c$ are considered to be interacting (cutoff is typically the radius of the network). The interaction potential V between the nodes is given by,

1 $$ V = {\displaystyle {\sum}_{jl}}\frac{1}{2}{K}_{jl}{\left({r}_{jl}-{r}_{jl}^0\right)}^2 $$

The spring constants are given by,

2 $$ \begin{array}{l}{\mathrm{K}}_{jl}=\gamma, \kern2em {r}_{jl}^0\le\ {R}_c\\ {}{\mathrm{K}}_{jl}=0,\kern2em {r}_{jl}^0>{R}_c\end{array} $$

An atomic analysis would entail considering the indices *j* and *l* run over all heavy atoms in the input protein structure, ${\boldsymbol{r}}_{\boldsymbol{jl}}^0$ being the distance between the coordinates (positional) of the nodes *j* and *l*. For a structure with N nodes interacting via the potential in eqn.1, only the force constant **γ** and the cutoff distance *R*_c_ need to be specified to construct the 3Nx3N dimensional Hessian. The Hessian is diagonalized to obtain 3 N-6 orthogonal eigenvectors (normal modes) and their eigenvalues (spring constants) [15]. The set of normal modes φ = {φ_**1**_, φ_**2**_ …. φ_**3N**-**6**_} are sorted in ascending order of their corresponding spring constants {*κ*_*1*_ < *κ*_*2*_ < ……*κ*_*3N*-**6**_} or in descending order of their variances $\left\{\sqrt{\frac{k_B T}{\kappa_1}} > \sqrt{\frac{k_B T}{\kappa_2}}>\dots \dots \sqrt{\frac{k_B T}{\kappa_{3 N-6}}}\right\}$. The spring constants are directly proportional to the square of mode frequencies. Lower spring constants therefore denote lower frequency modes and describe a more collective atomic motion.

The simplest implementation of uniform γ-value in the interatomic potential is used in this study and is set to unity (1.0 kcal/mol/Å^2^) for all interacting nodes within the network. The force constant uniformly scales the absolute amplitudes of atomic fluctuations. Since the relative values of spring constants for two proteins are studied here, only the relative size of the amplitudes is of interest and hence, γ-value is not varied here. The other parameter ${R}_c$ is considered to be variable in the potential for the atomic ANM-based calculations here. The major utility of the model is to predict the relative size/strength of deformation along a direction connecting an amino acid pair under tension.

ANM provides a collective mode description of equilibrium protein dynamics [16] which is primarily depicted through the slow (low-frequency) normal modes of the protein. In this paper, the harmonic model is used to provide a simple measure of protein stiffness in terms of spring constants obtained from the eigenvalues (${K}_i$) of the normal modes. An overall protein spring constant is defined as

3 $$ {k}_{protein}=\frac{k_B T}{{\left({\sigma}_{protein}\right)}^2} $$

The cumulative variance (${\sigma}_{protein}^2$) from the 3 N-6 ($={N}_{modes}$) modes, N being the number of heavy atoms is defined as

4 $$ {\sigma}_{protein}^2={\displaystyle {\sum}_{i=1}^{N_{modes}}}\left(\frac{k_B T}{\kappa_i}\right) $$

The variance in atomic fluctuations increases with the number of modes leading to a decrease in the overall protein spring constant. The value of ${k}_{protein}$ converges rapidly with the cumulative modes (${N}_{modes}\Big)$. The ${k}_{protein}$ measure represents an overall protein stiffness based on fluctuations of all (heavy) atoms in the protein.

If the normal modes are projected along a direction that coincides with the direction of deformation of the amino acid pair subjected to a pulling force, it can be estimated whether the modes contribute in favor of or against the force. Another measure of protein stiffness is defined as [12].

5 $$ {k}_{d irect}\left( m, n\right)=\frac{{\displaystyle {\sum}_{i=1}^{N_{modes}}}\left({\kappa}_i\ {d}_{mn}^i\right)}{{\displaystyle {\sum}_{i=1}^{N_{modes}}}{d}_{mn}^i} $$

This denotes the effective protein stiffness along a direction specified by the C_α_ atoms of two residues *m* and *n* and this represents the deformation direction. It becomes more relevant to correlate this quantity with the spring constants derived from unfolding forces (measured with single-molecule AFM experiments) along a pulling direction. The deformation of the protein along the distance vector joining the C_α_ atoms of residues *m* and *n* is given by,

6 $$ {d}_{mn}^i=\sqrt{\frac{k_B T}{K_i}}\left| cos\left({\theta}_{mn}^i\right)\right|\left|{\boldsymbol{u}}_{\boldsymbol{mn}}^{\boldsymbol{i}}\right| $$

and ${\boldsymbol{u}}_{\boldsymbol{mn}}^{\boldsymbol{i}}$ is the difference between the vectors generated from the atomic (C_α_) coordinates of *m* and *n* of the *i* ^*th*^ normal mode [12]. The projection of each normal mode along this direction is defined by the dot product as

7 $$ cos\left({\theta}_{mn}^i\right)=\frac{{\boldsymbol{r}}_{\boldsymbol{mn}}^0 \cdot \kern0.5em {\boldsymbol{u}}_{\boldsymbol{mn}}^{\boldsymbol{i}}}{\left|{\boldsymbol{r}}_{\boldsymbol{mn}}^0\left|\right|{\boldsymbol{u}}_{\boldsymbol{mn}}^{\boldsymbol{i}}\right|} $$

The equilibrium distance ${\boldsymbol{r}}_{\boldsymbol{mn}}^0$ is a vector between the C_α_ atoms of *m* and *n*, as obtained from their position coordinates in the input X-ray crystal structure. The magnitude (absolute value) of the dot product is considered in Equation 6 because this formalism only requires a set of eigenvectors (normal modes) that are orthogonal to each other. The phase between the modes and hence, the eigenvector direction is arbitrary. The overall spring constant ${k}_{protein}$ and the effective spring constant ${k}_{direct}$ are the observables which are computed for the structurally similar FN domains in this paper.

For atomistic ANM-based calculations, the cutoff distances ${R}_c$ used are typically around 5 Å or even smaller [17]. Since there is no general consensus on this parameter, the spring constant values are computed for different cut off distances and consequently, the selection of a cutoff ${R}_c$ is justified to yield an optimal agreement between theoretically and experimentally achieved spring constant values. The ProDy software program [18] has been used for normal mode calculations on the X-ray crystal structure of human fibronectin protein (PDB: 1fnh) that binds to heparin and integrin [13]. The analysis done here establishes protein stiffness as a molecular descriptor that seems to be highly sensitive to the sequence of structurally equivalent proteins.

## Results and Discussions

**Fig. 1:**
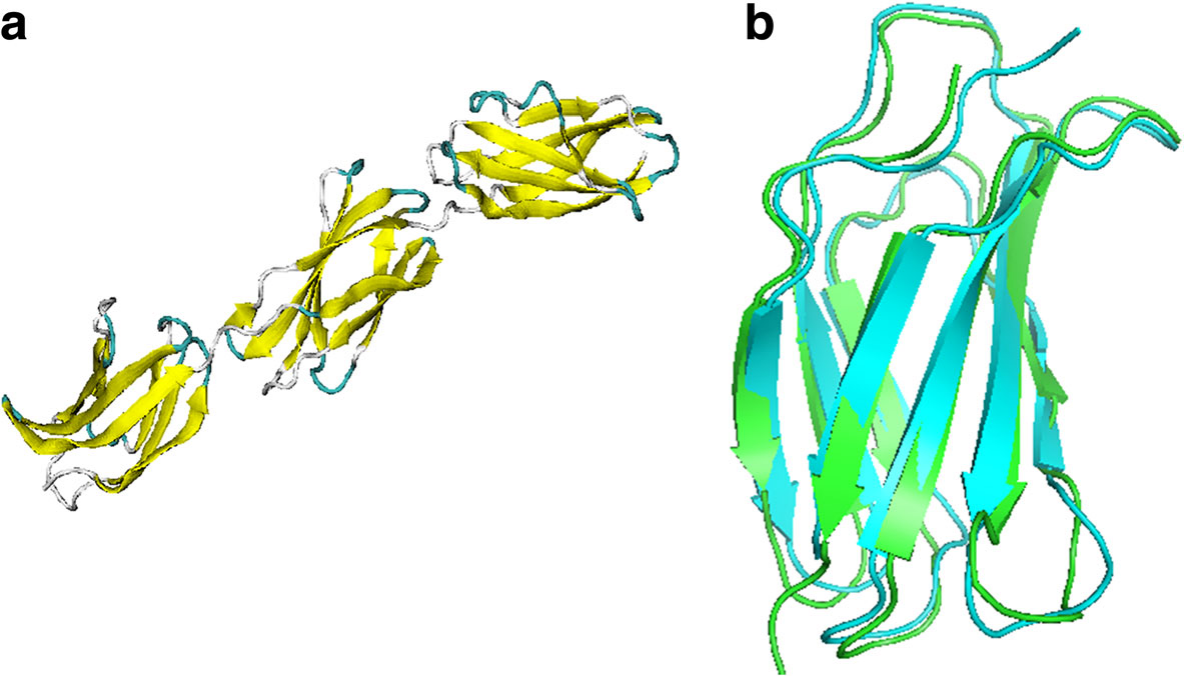
**a** Crystal structure of the heparin- and integrin-binding segment of human fibronectin protein (PDB: 1fnh) (**b**) Backbone superposition of the two domains, ${\mathrm{FNIII}}^{12}$ (FN1 is *green*) and ${\mathrm{FNIII}}^{13}$ (FN2 is *blue*)

Fibronectin mediates a number of cellular interactions with the extracellular matrix and has vital roles in cell adhesion, migration, growth and differentiation [19]. The type-III repeats of FN are the two structurally homologous domains which exhibit different mechanical responses to deformations along their N-C termini directions, owing to the difference in their sequences [14]. These domains are around 90 amino acids long and the one ${\mathrm{FNIII}}^{12}$ having residue indices 3–92 (PDB: 1fnh, X-ray crystal) requires a higher unfolding force than the other ${\mathrm{FNIII}}^{13}$ having residue indices 93 – 181. The former is the most stable/strongest domain in FN and the latter is the weakest, indicating important roles of individual domains in the activation of fibrillogenesis and matrix assembly [19]. Unfolding forces demonstrated in [14] are 125 pN and 89 pN for ${\mathrm{FNIII}}^{12}$ and ${\mathrm{FNIII}}^{13}$ respectively (the former is represented as FN1 and the latter as FN2 in this paper). A quantitative description of the stiffness of these domains (in their native states) can be given by the spring constant measures defined in the preceding section and one has to observe the correlation (if any) between the computed spring constants and the experimentally measured forces. Figure 1b shows that although they share only 25% sequence identity, yet the secondary structural elements of the two domains completely align with each other. The terminal residues of FN1 are proline (P3) and glutamate (E92) and that of FN2 are asparagine (N93) and threonine (T181) and the equilibrium distances between the C_α_ atoms of these amino acid pairs in FN1 and FN2 form the respective pulling directions.

**Fig. 2:**
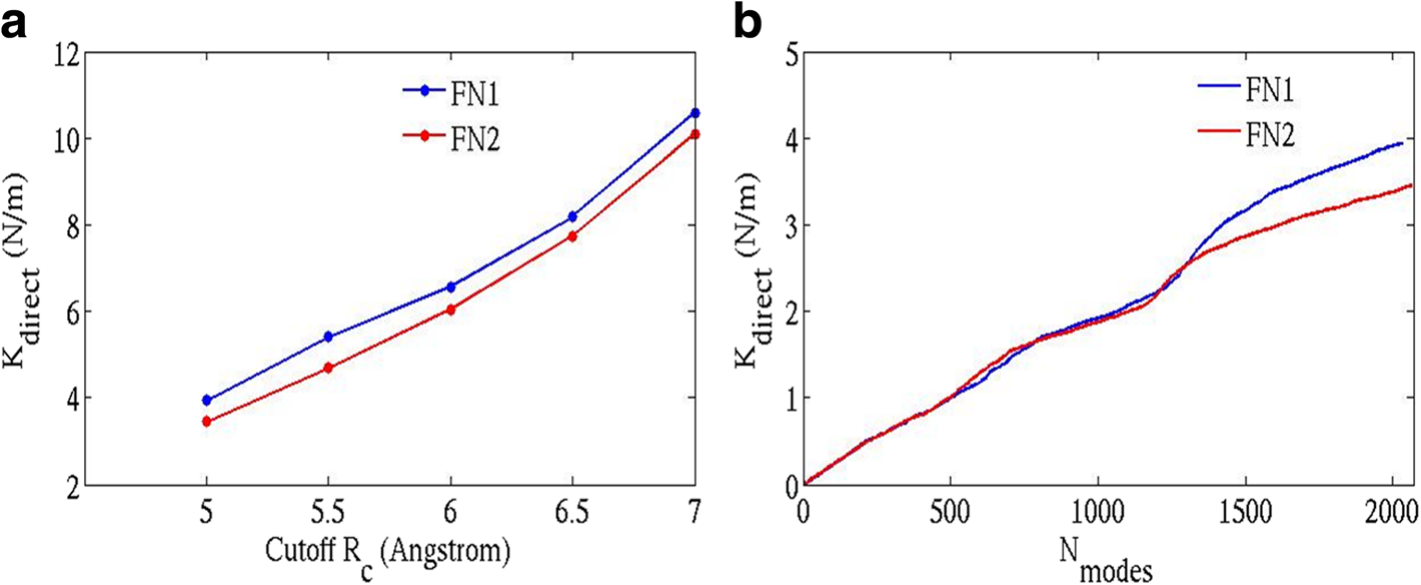
**a** Plot of ${k}_{direct}$ *versus* cutoff distance. The minimum cutoff that can be used for both the fibronectin crystal structures is ${R}_c$ = 4.8 Å (below this, more than six zero eigenvalues are obtained). Hence, cutoffs from 5.0 Å have been shown here (**b**) Plot of ${k}_{direct}$ *versus* cumulative normal modes $\left({\mathrm{N}}_{\mathrm{modes}}\right)$, for the two fibronectin domains ${\mathrm{FNIII}}^{12}$ and ${\mathrm{FNIII}}^{13}$ (named as FN1 and FN2 respectively) at ${\boldsymbol{R}}_{\boldsymbol{c}}$ = **5.0 Å**. FN1 has 680 heavy atoms (${\mathrm{N}}_{\mathrm{modes}}$ =2034) and FN2 has 691 heavy atoms (${\mathrm{N}}_{\mathrm{modes}}$ =2067)

**Fig. 3:**
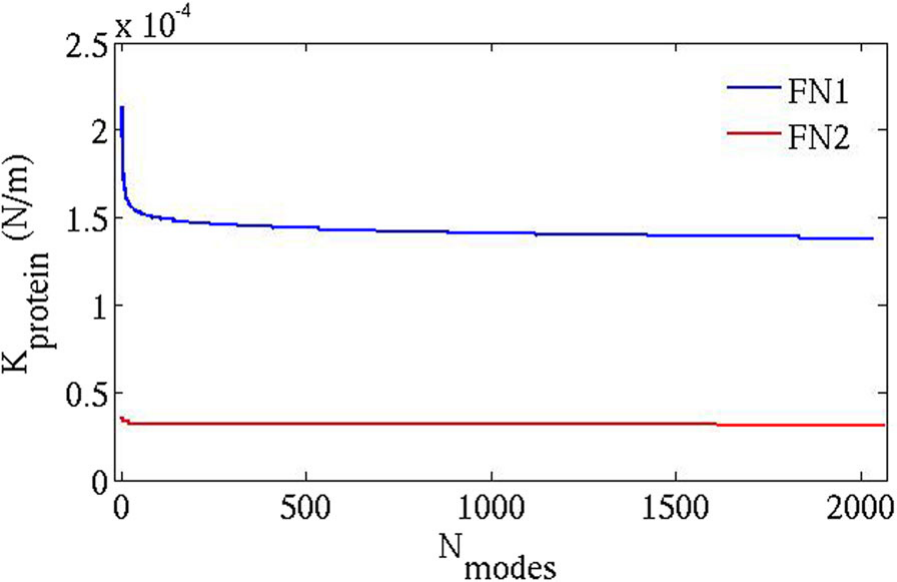
Plot of ${k}_{protein}$ *versus* cumulative normal modes (${\mathrm{N}}_{\mathrm{modes}}\Big)$, for both the fibronectin domains ${\mathrm{FNIII}}^{12}$ and ${\mathrm{FNIII}}^{13}$ (named as FN1 and FN2 respectively) at ${\boldsymbol{R}}_{\boldsymbol{c}}$ =**5.0 Å**

Since I build upon a previous approach by Eyal and Bahar [12] by inclusion of all heavy atoms in ANM, I discuss the directional (N-C termini) stiffness of the proteins domains followed by the overall stiffness. Figure 2a illustrates the relative effective stiffness (given by ${k}_{direct}$ in N/m) of the two structures FN1 and FN2 (along their N-C termini, P3-E92 and N93-T181 respectively) as a function of the cutoff ` ${R}_c^{\prime }$. A wide range of cutoff distances (as low as 5 Å and as high as 7 Å) have been examined (Fig. 2a). The FN1 domain remains stiffer than the FN2 domain implying that the normal modes are less ‘yielding’ in FN1, as a consequence of which relatively stronger external force is required to induce a similar deformation in FN1 as in FN2. The relative trend of ${k}_{direct}$ values remains consistent in the entire range of cutoffs (5.0 Å to 7.0 Å) and hence, any cutoff value in this window can be explored to test the measures of protein stiffness. The cutoff distance ${R}_c$ =5.0 Å is chosen hereafter in this study to examine the effective spring constant (${k}_{direct}$) values against the cumulative normal modes (shown in Fig. 2b). It depicts that FN1 is stiffer than FN2 but is only clearly evident at the higher frequency modes. It is quite likely that apart from contributions of the low frequency modes, the high frequency modes that represent localized motions of a small group of atoms (e.g., protein side-chains) also contribute towards deformations in both FN1 and FN2 considerably. The effective spring constant ${k}_{direct}$ is an anisotropic property of the protein. This measure depends on two quantities viz., (i) the distance between two residues and (ii) the length (in Å) to which each normal mode (slow or fast) projects onto the direction defined by this distance. The atomic fluctuations of high amplitudes (side-chains of residues in most cases) that arise from such fast motions (at close proximity to this direction/distance) might as well project to a large extent and lower the internal resistance to the deforming force externally applied along this distance/direction. Hence, it is necessary to consider all (low- and high-frequency) modes (instead of only the slowest) in computing ${k}_{direct}$ for a protein structure. The high-frequency modes of FN2 make a more significant contribution in lowering the internal resistance to external force than that of FN1 and hence, a smaller force is required to induce a deformation along the N-C termini direction of FN2. As the modes are sorted in increasing order of spring constant (decreasing order of variance), the ${k}_{protein}$ value converges rapidly with the cumulative modes (evident from Fig. 3). Therefore, only top few (low-frequency) modes that represent global atomic motion at equilibrium are sufficient to capture relative trends in protein flexibility. The ${k}_{protein}$ value is an average measure and reflects an overall breathing motion of the protein at equilibrium.

The ${k}_{protein}$ measure describes the compactness (packing fraction) of the protein and is dictated by the number of interacting atomic pairs enclosed within the network. The compactness and therefore, the flexibility primarily arise from short-range interactions in the protein and a higher cutoff would push the system towards physically unreasonable interactions (longer-range) in interpreting the overall protein flexibility. It is thus a reasonable choice to stick to smaller cutoffs in order to precisely estimate the protein-stiffness measure. To compute the overall spring constant $\Big({k}_{protein}$) for the protein domains considered (shown in Fig. 3), the same cutoff ${R}_c$ =5.0 Å hence appears reliable. The FN1 domain is stiffer (more compact) than the FN2 domain and this feature is already captured by the top few modes which represent the slow (low-frequency) modes.

For a more quantitative/numerical comparison of the theoretical results and the published experimental results, a table (Table 1) is provided. This shows the unfolding forces deduced from experimental measurements [14] and the corresponding ${k}_{direct}$ values and ${k}_{protein}$ values obtained from this theoretical work on the fibronectin domains FN1 and FN2. There is definitely a positive correlation between the overall flexibility and directional flexibility, also between each of these and the experimentally observed unfolding forces of the globular protein domains considered.

**Table 1:** Comparison of the theoretical results and experimentally reported pulling forces for the fibronectin domains

Proteins	Unfolding forces in pN (single-molecule AFM published studies)	${k}_{direct}$ in N/m (theoretical studies here)	${k}_{protein}$ in N/m (theoretical studies here)
FN1	125	3.9	1.4 × 10^− 4^
FN2	89	3.4	0.3 × 10^− 4^

An all-atom ANM incorporates the sequence information (amino acid composition) of the proteins and therefore, it is suitable for studying the mechanical flexibility of proteins that share the same tertiary fold. For the FN domains, the spring constant values (characterizing protein stiffness/flexibility) correlate well with the effective unfolding forces observed experimentally. The computational framework provided here can be explored in conjunction with single-molecule AFM experiments on proteins (in which sequence dominates over topology in describing mechanical stability) to probe the role of native stiffness in controlling the diverse range of cellular processes mediated by such proteins.

## Conclusions

The atomic level ANM presented here reveals the utility of studying equilibrium protein dynamics which is intrinsically favored by their native folds from straightforward calculations. The estimation of protein stiffness from the two different spring-constant measures elaborated in this paper is computationally efficient and readily available for experimental tests. The effective stiffness yields an account of the anisotropic mechanical behavior of the protein which is dictated by the normal modes, helping to assess the kinetic accessibility along a certain direction in the structure. In this study, the focus completely lies on relative values of spring constants that characterize the stiffness of proteins. The theory explains the differences in flexibility of proteins (close to their native states) that share the same fold and is presumably a powerful tool in predicting the higher mechanical strength of one protein over the other. Since this theoretical framework is based on the evaluation of structural data obtained from the protein databank (X-ray or NMR structure), the framework can be extended to study the native-state characteristics of a database of proteins that differ widely in sequence yet have structural homology, and guide the experimental researchers in designing single-molecule AFM pulling experiments (selection of residues along which pulling forces should be applied).

The all-atom model utilizes protein native-state coordinates to predict the relative intrinsic flexibility of structurally homologous proteins. Events (non-native) far away from equilibrium are beyond the applicability of the model. The model appears to be able to predict correlations between the theoretical spring-constant measures and the spring constants computed from experimentally measured unfolding forces of proteins. In addition, it recognizes the importance of sequence in cases like the FN domains and emphasizes the crucial role of sequence (over topology) in the mechanical responses of proteins.

## Abbreviations

AFM: Atomic force microscopy
ANM: Anisotropic network model
FN: Fibronectin
MD: Molecular dynamics
